# Role of glycosyltransferases in carcinogenesis; growth factor signaling and EMT/MET programs

**DOI:** 10.1007/s10719-022-10041-3

**Published:** 2022-01-28

**Authors:** Motoko Takahashi, Yoshihiro Hasegawa, Kento Maeda, Masato Kitano, Naoyuki Taniguchi

**Affiliations:** 1 Department of Biochemistry, Sapporo Medical University School of Medicine, South-1 West-17, Chuo-ku, Hokkaido, 060-8556 Japan; 2 Department of Glyco-Oncology and Medical Biochemistry, Osaka International Cancer Institute, 3-1-69 Otemae, Chuo-ku, Osaka, 541-8567 Japan

**Keywords:** Cell surface receptor, Collectin, EGFR, EMT programs, ErbB receptors, Fut8, GM3, GnT-III, GnT-V, ST6Gal1

## Abstract

The glycosylation of cell surface receptors has been shown to regulate each step of signal transduction, including receptor trafficking to the cell surface, ligand binding, dimerization, phosphorylation, and endocytosis. In this review we focus on the role of glycosyltransferases that are involved in the modification of N-glycans, such as the effect of branching and elongation in signaling by various cell surface receptors. In addition, the role of those enzymes in the EMT/MET programs, as related to differentiation and cancer development, progress and therapy resistance is discussed.

## Overview of studies of N-glycans of membrane proteins

Most secreted proteins and membrane proteins in eukaryotic cells are glycosylated, indicating that glycosylation is important for the regulation of signal transduction [1–8]. Glycans control many of the physicochemical properties of proteins, including structure, structural stability, charge, and hydrophilicity. In the case of cell surface receptors, glycosylation has been shown to regulate each step in signal transduction such as receptor trafficking to the cell surface, ligand binding, dimerization, phosphorylation, and endocytosis [9, 10]. To elucidate the mechanisms by which glycans control the function of these receptors, the site and the structure of the responsible glycan(s) need to be determined. To determine the site of the responsible glycan(s), a mutant receptor which lacks specific glycan(s) are used. The N-glycan deleted mutant receptor is established and the effects of the alteration of glycosylation site(s) on the function of the receptor can then be evaluated. For determining the structure of the responsible glycan(s), mass spectrometry analysis enables us to evaluate both the occupancy and glycan structure on each glycosylation site. By using these approaches, the function and the structure of a specific glycan can be determined. Determining glycan structure is especially important when lectin or lectin-like molecules are involved in the regulation of glycoproteins. However, it is nearly impossible to manipulate the glycan structure of a specific glycoprotein in a living cell, therefore, it is difficult to confirm the indispensability of certain glycan structures in a specific molecule. Collecting data related to the glycan function of a large number of membrane proteins might provide a clue for elucidating the roles of specific glycan structures. This review summarizes progress that has been made concerning the glycosylation of cell surface receptors.

## N-glycans of ErbB receptors

**Fig. 1:**
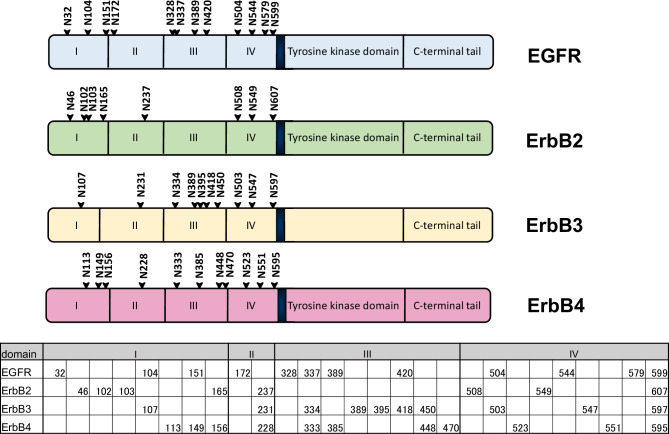
N-glycosylation sites of ErbB receptors. The upper panel indicates the schematic diagram showing the structural protein domain and N-glycosylation sites of ErbB receptors. The amino acid numbering is for the mature form of the receptors and does not include signal peptides of the N-terminal 24 amino acids of EGFR, the 22 amino acids of ErbB2, the 19 amino acids of ErbB3, or the 25 amino acids of ErbB4. The lower panel indicates the alignment of glycosylation sites of ErbB receptors

The ErbB family includes four members; EGFR (ErbB1, HER1), ErbB2 (HER2), ErbB3 (HER3), and ErbB4 (HER4). They are involved in a variety of biological events and their aberrant signaling has been implicated in the pathogenesis of various types of cancers [11, 12]. The ErbB receptors are classified as type I transmembrane receptor tyrosine kinases, and consist of an N*-*terminal extracellular domain, a transmembrane domain, an intracellular tyrosine kinase domain, and a C-terminal tail (Fig. 1). The binding of a ligand to the extracellular domain induces a conformational change from a “tethered form (the inactive form)” to an “extended form (the active form)” in which the dimerization arm mediates homo or heterodimers [13]. The receptor dimerization induces the phosphorylation of the tyrosine residues in the C-terminal tail, which subsequently activates the downstream signaling such as the PI3K/Akt pathway or the Ras/Erk pathway.

ErbB receptors are highly glycosylated. EGFR, ErbB2, ErbB3, and ErbB4 contain 12, 8, 10, and 11 N-glycosylation sites, respectively, in their extracellular domains. Figure 1 indicates the N-glycosylation sites of ErbB receptors, and the alignment of glycosylation sites of ErbB receptors, and the results indicate that some glycosylation sites are common among the four ErbB receptors.

### N-glycans of EGFR

**Fig. 2:**
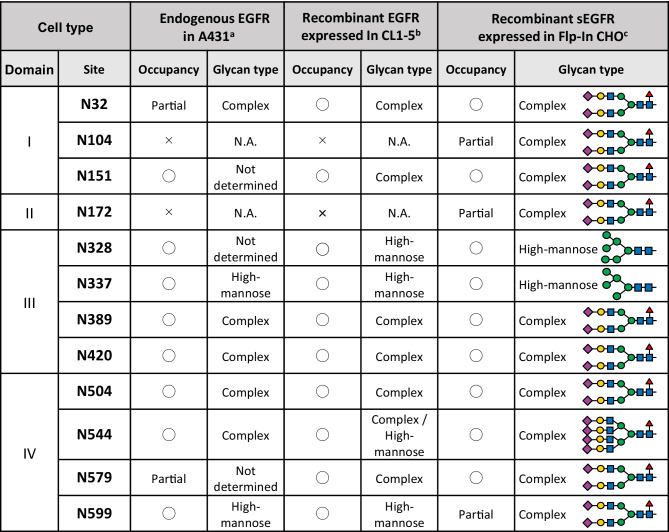
Site specific N-glycosylation status of EGFR. For each category (endogenous EGFR in A431, recombinant EGFR expressed in CL1-5, and recombinant sEGFR expressed in CHO-K1), the left column indicates glycosylation occupancy and the right column indicates the type of N-glycan. In the glycosylation occupancy columns, ◯ indicates 100% glycosylation and × indicates no glycosylation. ^a^Data from Zhen *et al*. [15]. The data of glycosylation status are common to full length EGFR and 105 kDa sEGFR, but the data of the N-glycan structure are of 105 kDa sEGFR. ^b^Data from Liu *et al*. [16]. 100% glycosylation and partial glycosylation are not discriminated here. ^c^Data from Hasegawa *et al*. [17]

EGFR is a ~ 170 kDa protein with 11 typical (N-X-S/T, where X is any amino acid except proline) and 4 atypical (N-X-C) N-glycosylation consensus sequences [14]. Figure 2 indicates the occupancy and glycan structure on each N-glycosylation site of endogenous EGFR in A431 human epidermoid carcinoma cells [15], recombinant EGFR, which is expressed in CL-1 human lung cancer cells [16], and recombinant soluble EGFR (sEGFR, the extracellular domain of EGFR) expressed in CHO-K1 cells [17]. In the case of sEGFR in CHO-K1 cells, it was observed that all 11 typical N-glycosylation consensus sequences (N104, N151, N172, N328, N337, N389, N420, N504, N544, N579, N599) are either fully or partially glycosylated, and one of the four atypical N-glycosylation consensus sequences (N32) is fully glycosylated. It should also be noted that the occupancy and glycan structures are well conserved in all cell types that have been examined to date.

The N-glycosylation of EGFR is required for ligand binding [18, 19], and the processing of oligosaccharides from high-mannose type to complex type does not affect this ability [20]. A site specific study has demonstrated that the N-glycan on N420 of EGFR is involved in dimerization [21]; among the four N-glycan deletion mutants of EGFR in which the glycosylation sites in domain III are mutated, the EGFR N420Q mutant exhibited ligand-independent oligomerization and phosphorylation. Another study reported that the deletion of the N-glycan on N579, which is in the domain IV auto-inhibitory tether loop, weakens the interaction between domains II and IV, thus increasing the ratio of high affinity binding receptors and also increasing the extent of ligand-independent dimerization [22]. These investigators assumed that N-glycan attached to N579 contributes to the stability of the inactive form, and that the deletion of the N-glycan might increase the structural flexibility of the moleculs. These studies suggest that specific N-glycans might be involved in stabilizing structure of EGFR to prevent unnecessary activation. Molecular dynamics simulation studies have also demonstrated that N-glycans are determinants of EGFR conformation, including the orientation of the extracellular domain relative to the membrane [23].

Glycan structures are also important for regulating EGFR when lectins or lectin-like molecules are involved. For example, in a previous study, we reported that the pulmonary surfactant protein D (SP-D) downregulates EGF signaling in lung adenocarcinoma [17, 24]. SP-D is an apoprotein of a pulmonary surfactant, belongs to the collectin subgroup of the C-type lectin superfamily which recognizes high-mannose type N-glycans in a calcium dependent manner [25, 26]. We have reported that SP-D directly binds to the N-glycans of EGFR, and downregulates the binding of EGF to EGFR and downstream signaling in human lung adenocarcinoma cells [17, 24]. As shown in Fig. 2, EGFR contains high-mannose type N-glycans at N328 and N337, and SP-D possibly binds to these glycans. It is speculated that the binding of SP-D to EGFR directly interferes with EGF binding, or that SP-D affects the conformation of EGFR, thus altering the ligand binding characteristics of the molecule.

It has been reported that EGFR with poly-*N*-acetyllactosamine (poly-LacNAc) containing N-glycans, which is produced by the enzymatic activity of *N*-acetylglucosaminyltransferase V (GnT-V), avoids constitutive endocytosis [27]. It has been proposed that galectin-3 binds to poly-LacNAc on the glycans of EGFR to form a molecular lattice, leading to the cell surface expression of EGFR being sustained. Intriguingly, it has been observed that the endocytosis of EGFR is increased in *N*-acetylglucosaminyltransferase III (GnT-III) transfected HeLaS3 cells [28]. GnT-III catalyzes the introduction of a GlcNAc unit to produce a “bisecting GlcNAc” structure [29], which prevents the formation of poly-LacNAc. It is possible that the binding of N-glycans of EGFR to galectin-3 is decreased by the activity of GnT-III, resulting in the upregulation of EGFR endocytosis. A case can be made that specific structures of N-glycans may regulate EGFR endocytosis through interactions with galectin-3.

The α1,6-fucosylation of N-glycans by the activity of α1,6 fucosyltransferase (Fut8) has been shown to affect EGFR function [30]. Fut8 catalyzes the addition of a fucose unit to the innermost GlcNAc residue of N-glycans, to produce α1,6-fucosylation, or a "core fucose" [31]. We previously reported that the loss of α1,6-fucosylation of N-glycans of EGFR reduces the binding of EGF to EGFR, and subsequent downstream signaling [30]. It has also been suggested that the increased sialylation and α1,3 fucosylation of the N-glycans of EGFR suppress EGF-induced EGFR dimerization [16, 32].

Hakomori *et al*. demonstrated that glycosphingolipids modulate transmembrane signaling and indicated that glycosphingolipid enriched microdomains are signaling platforms [1, 2, 33, 34]. EGFR is also regulated by glycoshpingolipids. GM3 (NeuAcα3Galβ4Glcβ1Cer) has been shown to interact with EGFR and downregulate its activation [35–41]. The binding activity of GM3 is much higher than that for other gangliosides, such as GM2, GD3, GM4, GM1, GD1a, and GT1b [40]. It has been reported that GM3 interacts with complex-type N-glycans with multivalent GlcNAc termini through carbohydrate-to-carbohydrate interactions (CCI). GM3 binds to the N-glycans of EGFR and inhibits the activation of tyrosine kinase and subsequent downstream signaling without affecting ligand binding activity [42, 43]. GM3 also inhibits EGFR tyrosine kinase activity by interacting with a membrane proximal lysine residue, K642 [44]. It has also been demonstrated that membrane-associated sialidase NEU3, whose selective substrates are GM3 and GD1a [45], activates EGFR [46]. It has been reported that GM3 is involved in the development of cancer [47–49].

### N-glycans of ErbB3

ErbB3 is a receptor for neuregulin1 (heregulin), neuregulin 2, and neuregulin 6. The unique character of ErbB3 is its lack of tyrosine kinase activity [50]. Therefore, ErbB3 forms a heterodimer with other ErbB receptors and exerts downstream signaling, such as the PI3K/Akt pathway or Ras/Erk pathway. The activation of PI3K/Akt signaling has been implicated in the ErbB3-dependent progression of various types of cancer [51–53]. The induction of ErbB3 expression or signaling is an important factor in drug resistance in several cancer models. Because of the above findings, ErbB3 is considered to be a promising target for cancer therapy [54, 55].

**Fig. 3:**
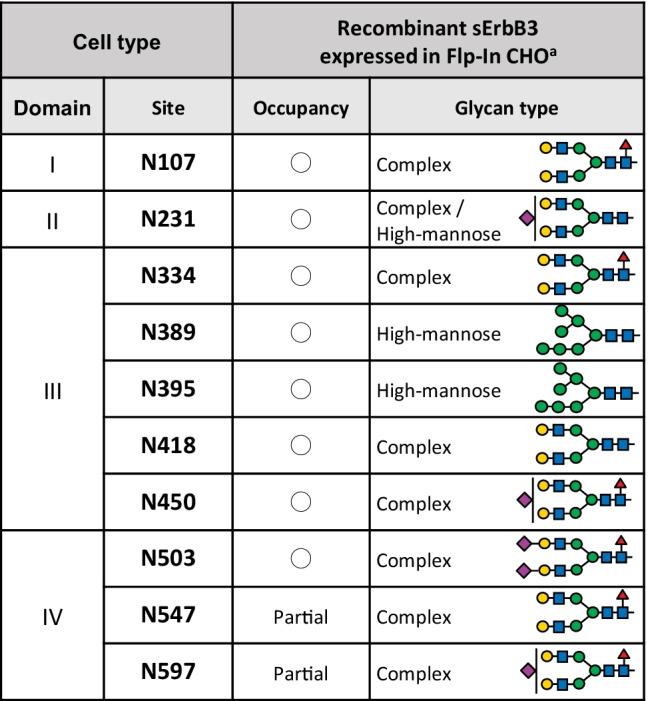
Site specific N-glycosylation status of ErbB3. In the glycosylation occupancy columns, ◯ indicates 100% glycosylation and × indicates no glycosylation. ^a^Data from Takahashi *et al*. (under submission)

ErbB3 is a ~ 185 kDa protein with 10 N-glycosylation consensus sequences in the extracellular domain. Figure 3 provides a summary of the occupancy and glycan structure on each N-glycosylation site of recombinant soluble ErbB3 that is expressed in CHO-K1 cells. The N-glycan on N418 of ErbB3, which corresponds to N420 of EGFR based on sequence alignment (Fig. 1), is involved in dimer formation. Among the 10 single N-glycan deletion mutants of ErbB3, the ErbB3 N418Q mutant forms a heterodimer with ErbB2 without ligand stimulation, exerts downstream signaling and promotes tumor formation in athymic mice [56]. These findings suggest that the N-glycans play a role in maintaining the inactive form of ErbB receptors in the absence of a ligand. It is possible that the conformational changes from the inactive form to the active form in the N-glycan deletion mutant of ErbB3 N418Q requires less energy [57].

The extracellular domain of ErbB3 (= soluble ErbB3, sErbB3) exerts suppressive effects on heregulin signaling, and these effects are enhanced in N418Q mutant [58, 59]. It is possible that the frequency of binding of the sErbB3 N418Q mutant to ErbB2 or other receptors on the cell surface is higher than that of the wild type [10].

## EMT/MET Programs and glycans of cell surface receptor

We previously reviewed the role of N-glycosylation of several cell surface receptors such as TGF-β receptor, RTKs, Integrins, Wnt, Hedgehog, Notch, and the involvement of glycans in inflammation and hypoxia-induced EMT (endothelial-mesenchymal transition) in various kinds of diseases [9, 60]. It is well known that phenotypic changes due to EMT play pivotal roles during embryonic development [61–64], wound healing [65], cancer and fibrosis [66]. Especially in cancer, invasion, metastasis and chemoresistance are considered to be highly associated with EMT [67]. The significance of EMT was also reported in COPD (chronic obstructive pulmonary disease) [68, 69], interstitial pneumonitis [70], and lung cancer [69]. Moreover, EMT has also been implicated in resistance to therapy in cancer [67, 71]. It has also been reported that EMT contributes to the development of resistance to the EGFR-tyrosine kinase inhibitor in non-small cell lung cancer [72]. The implication of COVID-19 infection has been also reported [73].

It was recently reported that an intermediate type of cell, referred to as a hybrid cell is produced between EMT and its reversive process, MET (mesenchymal-epithelial transition) [74–76]. A classical type of EMT programs were reported to be rather one-way processes, but recently it has become recognized that the EMT programs are more dynamic and can sometimes be reversed by epigenetic modification and gene regulation. There are distinct biomarkers for EMT and MET [77]. Among them, E-cadherin, claudin, occludin, and cytokeratin are typical examples because the gene expression patterns of these molecules are activated in MET and downregulated in EMT. On the other hand, N-cadherin, collagen, matrix metalloproteinases, fibronectin and vimentin are highly upregulated in EMT and downregulated in MET. There are also EMT transcription factors such as the Snail family referred to as SNAIL(SNAI1), SLUG (SNAI2) and SMUG (SNAI3), the basic helix-loop-helix protein family referred to as TWIST1 and TWIST2, and zing finger E-box binding transcription factors, referred to as ZEB1 and ZEB2 [67]. However, the issue of biomarkers for hybrid cells remains unclear at this time.

During EMT programs, the most important hallmark is TGF-β activation in various cancers. At the early stage of cancer, TGF-β acts as a protective factor whereas during carcinogenesis TGF-β functions as a progressive factor [78]. TGF-β is essential in developmental period as well. For example, TGF-β signaling facilitates the embryonic development of the lung and the aberrant glycosylation of TGF-β decreases signaling and downregulates the phosphorylation of Smad, thus causing emphysematous changes [79, 80].

**Fig. 4:**
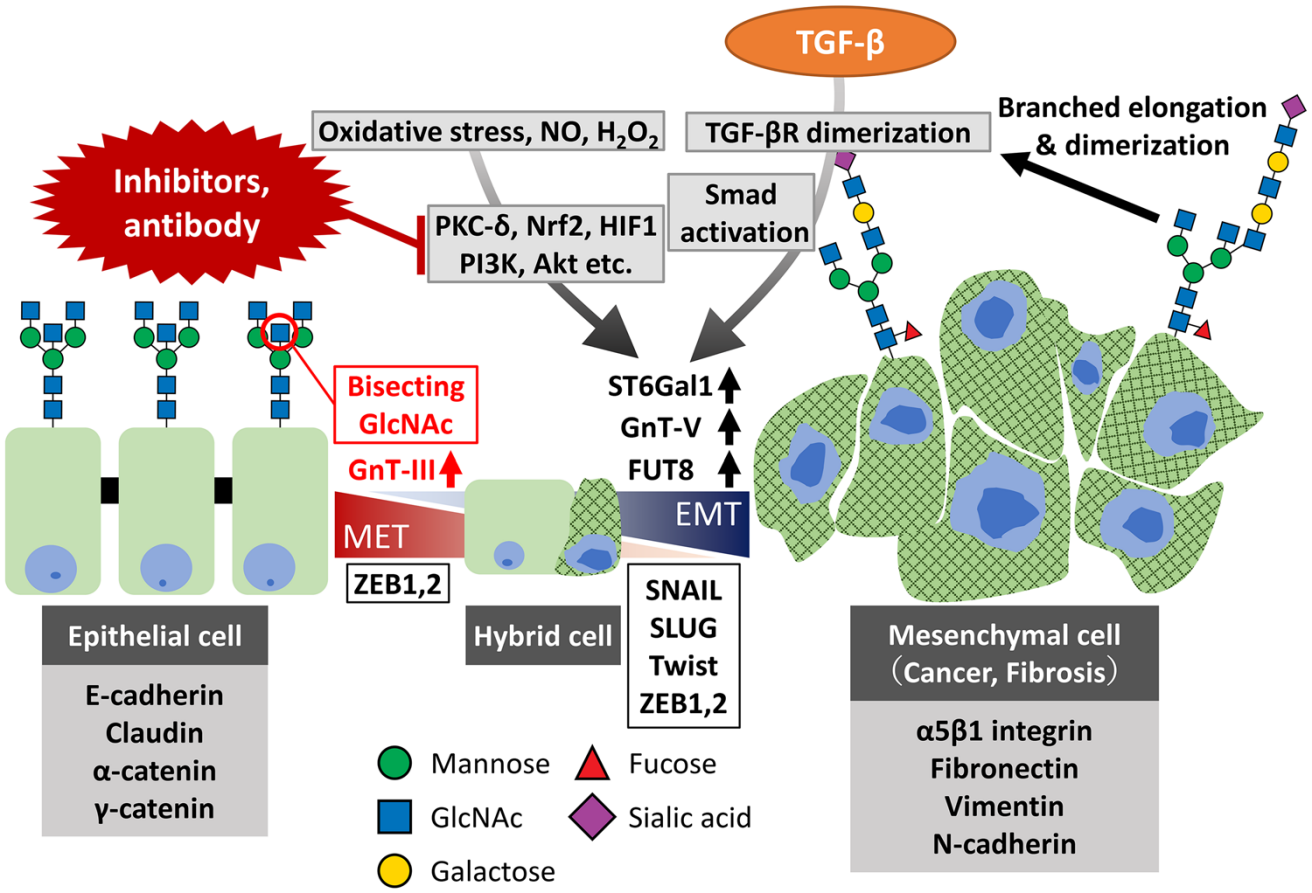
EMT/MET programs and changes in glycosyltransferases. Various effects of GnT-V, Fut8, ST6Gal1 and GnT-III in EMT/MET programs. GnT-III is implicated in the MET whereas ST6Gal1, GnT-V and Fut8 are implicated in EMT and it is possible that these changes are stimulated by various factors such as signaling molecules related to oxidative stress, via various signaling molecules including transcription factors such as ZEB1, 2, which are implicated in both EMT and MET

EGFR stimulates EMT during differentiation processes and apoptosis of cancer cells [81–83]. Resistance to therapy due to EGFR signaling has been reported [81]. Involvement of glycosphingolipids in EMT has also been demonstrated [84]. From these facts, changes in glycans in cell surface receptors may also play important roles in EMT and MET processes. Our group reported on the significance of branched N-glycans in various diseases, and in the case of EMT, our findings indicate that some glycosyltransferase genes of N-glycan branching and extension such as GnT-V, Fut8 and ST6Gal1 are upregulated in case of MET, whereas GnT-III is downregulated [85, 86] as shown in Fig. 4. However, the expressions of those genes may be sometimes upregulated in both EMT and MET, and probably at the intermediate stage, *i.e*. in the hybrid cells, some of these enzyme expressions are reversible [60, 87, 88]. Response to reduction–oxidation (redox) is one of the most important biological phenomena for maintaining homeostasis of the body under conditions of various types of oxidative stress [89] under pathophysiological conditions. Our group has been interested in redox regulation under conditions of oxidative stress as well as glycobiology in relation to disease [90–94]. Functional and structural changes in glycans are regulated by redox responses resulting from the generation of reactive oxygen species (ROS) or reactive nitrogen species (RNS) in various diseases including cancer [95], diabetes [96], neurodegenerative diseases [97], such as Parkinson’s disease, Alzheimer's disease as well as amyotrophic lateral sclerosis (ALS) (87), COPD [98] and aging. We proposed that the field "glyco-redox" investigations will open avenues to developing a more comprehensive understanding of the mechanism associated with diseases, as related to changes in glycan structures under oxidative stress [90]. The significance of this interplay was also reported by other groupes [99, 100]. It is well known that signaling molecules such as PI3K/Akt [101], PKC-delta [102], Nrf2 [103], HIF1 [104] and Smad [105] are closely associated with redox regulation. Some of these signaling molecules also regulate the activities of glycosyltransferases that are involved in N-glycan biosynthesis such as ST6Gal1 [106], GnT-V and Fut8, etc.. It has also been reported that some transcription factors that regulate GnT-III, V, and Fut8 as well as ST6Gal1 may play key roles in the intermediate states. In order to regulate the EMT programs for preventing cancer development, progression, metastasis and therapy resistance, specific antibodies or specific inhibitors against various transcription factors toward N-glycan glycosyltransferases are likely candidates for novel therapeutics in the future as shown in Fig. 4.

## Data Availability

The data that support the findings of this study are available from the corresponding author upon reasonable request.
